# Sialylated Cervical Mucins Inhibit the Activation of Neutrophils to Form Neutrophil Extracellular Traps in Bovine *in vitro* Model

**DOI:** 10.3389/fimmu.2019.02478

**Published:** 2019-11-06

**Authors:** Kim F. Bornhöfft, Alexander Rebl, Mary E. Gallagher, Torsten Viergutz, Kristina Zlatina, Colm Reid, Sebastian P. Galuska

**Affiliations:** ^1^Institute of Reproductive Biology, Leibniz Institute for Farm Animal Biology (FBN), Dummerstorf, Germany; ^2^Faculty of Medicine, Institute of Biochemistry, Justus Liebig University Giessen, Giessen, Germany; ^3^Institute of Genome Biology, Leibniz Institute for Farm Animal Biology (FBN), Dummerstorf, Germany; ^4^UCD Veterinary Sciences Centre, Dublin, Ireland

**Keywords:** neutrophil extracellular traps (NET), mucins, sialic acids, reproduction, sialic acid binding immunoglobulin-like lectins (siglecs), bovine neutrophils

## Abstract

In order to combat invading pathogens neutrophils can release neutrophil extracellular traps (NETs). However, since NETs can also damage endogenous cells, several control mechanisms for the formation of NETs must work effectively. For instance, neutrophil activation is silenced within blood circulation by the binding of sialylated glycoconjugates to sialic acid binding immunoglobulin-like lectins (Siglecs) on neutrophils. As neutrophils are recruited within the female reproductive tract, after mating, a comparable mechanism may also take place within the bovine cervix to prevent an exaggerated NET formation and thus, infertility. We examined, if the highly glycosylated mucins, which are the major functional fraction of biomolecules in mucus, represent a potential regulator of NET formation. The qPCR data revealed that in polymorphonuclear neutrophils (PMNs) inhibitory Siglecs are the most frequently expressed Siglecs and might be a potential target of sialylated glycans to modulate the activation of PMNs. Remarkably, the addition of bovine cervical mucins significantly inhibited the formation of NET, which had been induced in response to lipopolysaccharides (LPS) or a combination of phorbol myristate acetate (PMA) and ionomycin. The inhibitory effects were independent of the stage of estrous cycle (estrus, luteal, and follicular mucins). PMNs retained their segmented nuclei and membrane perforation was prevented. However, the inhibitory effects were diminished, when sialic acids were released under acidic conditions. Comparable results were achieved, when sialic acids were targeted by neuraminidase digestion, indicating a sialic acid dependent inhibition of NET release. Thus, bovine cervical mucins have an anti-inflammatory capability to modulate NET formation and might be further immunomodulatory biomolecules that support fertility.

## Introduction

When sperm enter the female reproductive tract an immune response is initiated and polymorphonuclear neutrophils (PMNs) are recruited (1, 2). There, several defense mechanisms are initiated such as phagocytosis (3). Furthermore, the formation of neutrophil extracellular traps (NETs) can be activated (1, 2). These molecular traps are mainly targeted against invading pathogens and their mechanism of action includes trapping and killing instruments (4–6). The rapidly released chromatin meshwork traps viruses, fungi, and bacteria. Since the DNA is loaded with high amounts of antimicrobial biomolecules, such as histones, lactoferrin, neutrophil elastases, and antimicrobial peptides, the trapped pathogens can be efficiently eliminated. However, several of these antimicrobial molecules are also toxic for endogenous cells. Extracellular histones play a predominant role in this context (7). Interestingly, an exaggerated formation of NET and its adverse effects were reported during several diseases (5, 8–11). Besides life-threatening diseases such as sepsis, thrombosis and acute lung failure, it is likely that NET formation can affect the reproductive system influencing infertility, preeclampsia, and fetal loss (2). Thus, throughout the body a tight control of neutrophil activation is required to regulate the NET response.

For instance, in circulation, sialylated glycans on erythrocytes seem to silence neutrophils in humans. The activation of neutrophils is suppressed immediately subsequent to the detection of these sialylated glycoconjugates by sialic acid binding immunoglobulin-like lectin-9 (Siglec-9) (12). Thus, in blood, sialic acid dependent mechanisms exist to prevent an excessive NET formation.

Possibly, similar to the circulatory system, the female reproductive tract also produces glycoconjugates to modulate the activation of neutrophils. The surface of the female reproductive tract is coated with large amounts of mucus that mainly consist of mucins (13–18). These are highly glycosylated glycoproteins and are essential to counteract infections, prevent dehydration, and both physical and chemical injury (13, 19). Besides secretory mucins, which are the main functional components in mucus, mucins can be anchored in cell membranes. Remarkably, the covalently linked *O*-glycans can typically comprise over 70% of the molecular mass. In addition to *O*-linked glycans, *N*-glycans are present on mucins. Both, *N*- and *O*-glycans are frequently terminated by sialic acid residues (20, 21) and may trigger the activation of inhibitory Siglecs on neutrophils. An inhibition of activation via Siglecs is possible, since inhibitory Siglecs contain intracellular tyrosine based inhibition motifs (ITIM), which mediate the inhibitory action. These immunomodulatory lectins are transmembrane receptors which are expressed in different immune cells, including PMNs, of vertebrates (22).

Similar to sialylated glyconjugates in circulation, sialylated cervical mucins may exert an inhibitory effect on the activation of PMNs in the female reproductive tract. To address this hypothesis, we isolated bovine cervical mucins and tested their capability to inhibit the activation of NET formation. Since the glycosylation of cervical mucins can change during estrous cycle (14, 16), mucins were collected from estrus, luteal, and follicular stage samples. In addition to the native form, mucins decorated with chemically modified sialic acid residues and de-sialylated mucins were employed allowing an assessment of the biological impact of these sugar residues on the activation of PMNs.

## Materials and Methods

All reagents used were of analytical grade.

### Isolation of Bovine PMNs

The isolation procedure for bovine PMNs was based on the protocol of Schuberth et al. (23). Minor changes, as described in the following, were implemented. For the isolation of bovine neutrophils, fresh ethylenediaminetetraacetic acid (EDTA) -blood samples of normally cycling Holstein cows (7 months up to 7 years of age, female animals) were diluted 1:1 with 1 × sterile phosphate buffered saline (PBS) and the diluted samples were transferred to a layer of histopaque 1,077 (Sigma Aldrich, St. Louis, Missouri, USA) with a volume equal to the amount of fresh blood used. Following this, the samples were centrifuged at 1,280 × g for 30 min at 10°C without break. Based on the density of the cells, lymphocytes and monocytes as well as some thrombocytes are localized in the interphase between the plasma and the histopaque, whereas the PMNs are co-localized with the erythrocytes. Therefore, in the next step, the supernatant was removed. Subsequently, a hypotonic lysis was performed. Ten milliliter of sterile water was added to 5 mL of blood sample for 10 s then 10 mL of 2 × PBS was added to re-generate isotonic conditions. After a further centrifugation step (500 × g, 10 min, 4°C), hypotonic lysis was repeated followed by centrifugation at 220 × g for 10 min at 4°C. Finally, the pellet was washed twice with 1 × PBS and the cells were resuspended and counted in RPMI 1640 (Thermo Fisher Scientific, Waltham, USA) with 1% penicillin/streptomycin (PenStrep, Thermo Fisher Scientific) without the addition of fetal bovine serum. PMNs were isolated from blood received from the slaughterhouse or from the remaining blood samples of regulatory blood collections from other projects (7221.3-1-010/16-1). Both the blood sampling and the slaughter processes were performed in accordance with the guidelines, applicable laws and provisions for ethical regulations.

### Quantification of the Siglec-Expression

Real- Time quantitative Polymerase Chain Reaction (RT-qPCR) analyses were performed with the LightCycler 96 System (Roche, Basel, Switzerland) using the Sensi-FAST SYBR No-ROX Kit (Bioline, Luckenwalde, Germany). RNA was isolated by using the RNeasy Mini Kit (Qiagen, Hilden, Germany) including digestion with RNase-free DNase I for 15 min. The concentration and the purity of the isolated RNA were then checked with the NanoDrop 1000 spectrophotometer (NanoDrop Technologies, Thermo Fisher Scientific). Agarose gel electrophoresis validated the presence of intact 18S and 28S rRNA bands without genomic DNA contamination. For cDNA synthesis, 100 ng of RNA was individually reverse-transcribed in a total volume of 100 μL (final concentration 1 ng/μL) using the Super Script II kit (Thermo Fisher Scientific). A total cDNA equivalent of 5 ng RNA was used for copy number analysis. The qPCR program included an initial denaturation (95°C, 5 min.), followed by 40 cycles of denaturation (95°C, 5 min.), annealing (60°C, 15 s) and elongation (72°C, 15 s) steps and the fluorescence measurement (72°C, 10 s). No-template controls and positive controls (containing PCR-generated fragments of the target genes) were included to monitor contaminations and the accuracy of the detection, respectively. The used oligonucleotide primers amplified gene-specific products with lengths between 74 and 194 bp (Table 1). The performance of the chosen primers was checked before qPCR measurements via standard PCR and the resulting PCR products were sequenced. In addition, a standard curve was applied (*R*^2^ > 0.9999) and efficiency of the primers was verified. All primers utilized here had an efficiency score between 91.5 and 103.0%. The quality of the PCR products was assessed based on gel electrophoresis and melting-curve analysis. Light-Cycler data was analyzed using the LightCycler 96 analysis software v. 1.1; C_q_ values <35 were considered detectable. GAPDH (24), YWHAH, and RPL19 (25) were used as reference genes. For Siglec-8, two different primer pairs were used. In addition, PCR products were partly sequenced in order to confirm Siglec-8 expression. Sample values were normalized by calculating the GeoMean of the reference genes. The numbers of the individual Siglec transcripts were calculated as a percentage relative to the summed transcript numbers of all measured Siglec transcripts. The outlined method has been previously described (26).

**Table 1:** The primer pairs used for qPCR.

**Primer name (F: sense, R: anti-sense)**	**Sequence 5^**′**^ → 3^**′**^**	**NCBI accession code (nt. position)**	**Amplicon length (bp)**
cattle_siglec_1_F	GTATGAAGGGGTCCTGCTTCGT	XM_025001079 (354-376)	194
cattle_siglec_1_R	AGCTCAGGATTCCCCACCAT	XM_025001079 (547-527)	
cattle_siglec_2_F	TGAGGTTGGAGCCTGTGAAG	XM_010814857 (4028-4048)	74
cattle_siglec_2_R	CTCTGGCCCAGACGGTTG	XM_010814857 (4101-4083)	
cattle_siglec_3_F	CATCTTCTCCTGGACGTCAGC	XM_015458139 (488-508)	183
cattle_siglec_3_R	CGTGAAGCATAGGTGACATTGAG	XM_015458139 (670-648)	
cattle_siglec_4_F	CGCTTCAGCTTCCCTGATGAG	NM_001040570 (224-245)	140
cattle_siglec_4_R	TGCGTCCCTGGAAGCTCTC	NM_001040570 (363-344)	
cattle_siglec_5_F	GGGACCCCAGCAACAATGACT	XM_005219563 (460-481)	154
cattle_siglec_5 R	CGGGTTTCTCTCCTGTCACCT	XM_005219563 (613-592)	
cattle_siglec_8(I)_F	ACGTGCCCTGCTCCTTCT	XM_024978778 (543-560)	172
cattle_siglec_8(I)_R	TCCCCGAGGAGATGGAATC	XM_024978778 (714-696)	
cattle_siglec_8(II)_F	GCGGTCCTGGTAGCCATCTG	GGVB01041119 (1038-1057)	87
cattle_siglec_8(II)_R	TGTGCAGCACCTCACTAGGAG	GGVB01041119 (1124-1104)	
cattle_siglec_10_F	AGAAGGCCTGTGCATGGTCGT	NM_001206277 (286-307)	186
cattle_siglec_10_R	CGCCAAGGAGCTGGAATCGG	NM_001206277 (471-451)	
cattle_siglec_14_F	TGGATCTACTACACCAGGTGTG	XM_019978755 (1242-1263)	114
cattle_siglec_14_R	TCTGTCCTCTGGGCTCTGCAT	XM_019978755 (1355-1335)	
cattle_siglec_15_F	ATCGCCTAGAGCACCCAGTCA	NM_001192567 (869-889)	93
cattle_siglec_15_R	CCCGAGGGCTCATCTGGTTC	NM_001192567 (961-942)	

### Flow Cytometry

In order to determine the purity of the isolated bovine PMNs, flow cytometry was performed. Therefore, an aliquot of the isolated cells was taken, diluted in sterile PBS and placed in the Gallios, Beckman Coulter instrument. Granularity was examined by measuring the side scattered light and the forward scattered light as described previously in detail (27, 28).

### Isolation and Purification of Cervical Mucins

Bovine cervices were collected from animals sent to the abattoir for meat production. The stage of estrous cycle was determined by observation of gross ovary morphology (assessment of corpus luteum and follicle development), as well as cervix conformation and cervical mucus characteristics. Estrus samples were collected from reproductive tracts with clear mucus, a well-developed follicle as well as a regressing corpus luteum. Luteal samples were collected from tracts with ovaries showing a large and well-developed corpus luteum [stage 3 of luteal phase according to the classification of estrous stages by Ireland et al. (29)]. Follicular samples were collected from tracts showing no viscous mucus secretion and a well-developed follicle. Samples were stored on ice prior to mucus isolation. The first step of mucin purification was the repeated treatment of the tissue with 8 M guadinium hydrochloride (GdnCl) (Sigma Aldrich) in order to release the mucus over ~30 min. Then, after overnight rolling in at least an equal volume of GdnCl, Dithiothreitol (DTT) (final concentration 10 mM) was added and incubated for 5 h. Iodacetamide (final concentration 25 mM) was added and the samples were incubated overnight in the dark at room temperature (Rt). The isopycnic density gradient centrifugation was carried out in CsCl/GdnCl. The density of the samples was brought to 1.4 g/mL using solid CsCl and samples were placed in Beckman Ultra-clear tubes. Ultracentrifugation (Beckmann Coulter, Optima L-100 XP) took place at 65,000 rpm for 18 h at 10°C, using the 70 Ti rotor without break. Samples were then unpacked sequentially as 1 mL fractions and mucin containing samples were identified by taking 5 μL for blotting onto a polyvinylidine fluoride (PVDF) membrane using a Whatmann manifold Slotblot apparatus. The membrane was incubated in 1% periodic acid in 3% acetic acid for 30 min, washed twice with 0.1% Na metabisulphite in 1 mM HCl, then stained with Schiff's Reagent (VWR, Radnor, Pennsylvania, USA). 0.5 mL of each fraction was also weighed in order to calculate the density in g/ml. Samples rich in carbohydrate and above 1.35 g/mL were further pooled and loaded on a Sepharose CL-4B column (Sigma Aldrich) and eluted with 50 mM Tris/100 mM KCl, pH 7.5. Fractions of 4 mL were collected and those rich in carbohydrate were identified using Schiff's Reagent as before. Carbohydrate rich factions were pooled and freeze dried. In order to get rid of remaining buffer salts, the samples were dissolved in a small volume of water and loaded on a Biogel p6 column (Bio-Rad, Hercules, California, USA) for desalting. Fraction collection and mucin identification took place as described previously for the Sepharose CL4B column. As a final step samples were freeze dried and the quantity of purified mucins was determined by weighing. Purified mucins have a cobweb like appearance and are highly electrostatic. The purified samples of different cows were separately stored dry at −20°C in sealed cryotubes until required.

### NET Induction

After counting, 19,500 cells/well were seeded in a 12-well silicone chamber slide (Ibidi, Gräfelfing, Germany) in a total volume of 130 μL RPMI 1640 (containing 1% PenStrep) and incubated for 1 h at 37°C and 5% CO_2_ prior to NET induction. NET induction was performed by adding 1.5 μM phorbol myristate acetate (PMA) (Sigma Aldrich) in combination with 3 μM ionomycin (Cell Signaling, Danvers, Massachusetts, USA) for 4 h at 37°C and 5% CO_2_. In order to investigate the potential of bovine cervical mucins to inhibit the release of NET, NET was induced and different final concentrations of cervical mucins were applied: 1, 5, 10, 15 μg/μL as well as 20 μg/μL.

Further experiments were performed using hydrolyzed mucins, C7 modified mucins and neuraminidase digested mucins (final concentration each 20 μg/μL).

In addition to the combination of PMA and ionomycin, lipopolysaccharides (LPS) was used to induce NET formation. After counting 19,500 cells/well were seeded in a 12-well silicone chamber slide (Ibidi) in a total volume of 130 μL of RPMI 1640 medium supplemented with PenStrep and incubated for 1 h at 37°C and 5% CO_2_. The formation of NET was induced with LPS from *Pseudomonas aeruginosa* (Sigma Aldrich) using a final concentration of 20 μg/mL and again incubation took place for 4 h at 37°C with 5% CO_2._ In order to determine the potential of bovine cervical mucins to inhibit NET released induced by LPS, different final concentrations of cervical mucins were applied: 1, 5, 10, 15 μg/μL as well as 20 μg/μL.

To determine the pH of the medium, pH strips were used to monitor the pH throughout the experiments. No significant change of the pH was detectable over the duration, independent of the added substance (PMA, ionomycin, LPS, mucins).

### Nuclei Staining

After 4 h of stimulation, cells were fixed with 4% paraformaldehyde (PFA) for 30 min at 4°C. After washing several times, cells were permeabilized for 1 min with 0.5% Triton-X-100 followed by further washing steps. Subsequently, nuclear staining was performed with 4′,6-Diamidin-2-phenylindol (DAPI) (Carl Roth, Karlsruhe, Germany, 1 μg/mL) before a further fixation step with 2% PFA for 20 min at Rt took place. Samples were then mounted and analyzed using fluorescence microscopy (Zeiss Axio Imager A1, Carl Zeiss). The determination of the percentage of activated cells was based on the release of de-condensed DNA fibers during NETosis (DAPI staining). During NETosis neutrophil elastase and other neutrophilic granule proteins translocate to the nucleus, de-condensation of chromatin is triggered and the DNA is decorated by several granular proteins. The resulting chromatin swelling directly leads to cell rounding and rupture of the cell and release of the meshwork of chromatin, which is associated with granule proteins such as lactoferrin and neutrophil elastase (30). In contrast, apoptotic cells condensate their chromatin, the cell and its nucleus shrink and cell membrane blebs are formed (31). During necrosis cells lose their membrane integrity and the resulting influx of water and ions leads to swelling of the cytoplasm, the nucleus and the cell organelles. Subsequently, cell lysis and an uncontrolled release of cellular content such as proteins, partially degraded DNA and granules takes place. In contrast to NETosis, no initiated decoration of chromatin with granule proteins occurs and thus, no long DNA filaments are commonly visible, which are highly associated with granule proteins. Taken together, all cellular death occurs with a loss of the segmented nucleus structure and can be distinguished by their characteristic morphological changes of chromatin (32).

Total cell number was evaluated and the number of segmented nuclei was determined. Analysis was carried out on multiple (random) pictures of different biological samples.

### Immunofluorescence Staining

After 4 h of stimulation, cells were fixed with 4% PFA for 30 min at 4°C. After washing several times cells were permeabilized for 1 min with 0.5% Triton-X-100 followed by further washing steps. Blocking was performed with 2% IgG-free bovine serum albumin (BSA) for 30 min at 37°C. Subsequently, the antibodies, diluted in the blocking buffer, were added [rabbit pAb to Neutrophil Elastase (1:200; Abcam, Cambridge, Great Britain), goat anti-bovine Lactoferrin (final concentration 5 μg/mL, Biomol, Hamburg, Germany)]. Incubation of anti-Neutrophil Elastase took place overnight at 4°C, whereas the incubation of anti-bovine Lactoferrin took place for 1 h at 37°C. After the incubation of the first antibody, samples were washed and the secondary antibody was added. In order to detect neutrophil elastase Alexa Fluor 488 goat anti-rabbit IgG (final concentration 2 μg/mL, Invitrogen, Carlsbad, California, USA) was used and to detect Lactoferrin Alexa Fluor 568 donkey anti-goat IgG (H+L) was applied (final concentration 2 μg/mL, Molecular Probes, Eugene, Oregon, USA). Incubation took place for 1 h at 37°C (Alexa Fluor 568) or for 1 h at Rt (Alexa Fluor 488). Afterwards a further washing step was performed and subsequently, nuclear staining was performed with DAPI (Carl Roth, 1 μg/mL) before a fixation with 2% PFA for 20 min at Rt took place. Samples were then mounted and analyzed using fluorescence microscopy (Zeiss Axio Imager A1, Carl Zeiss). For each stage of estrus cycle, neutrophils from three independent cell isolations (2 different animals) were used. Total cell number and cells positive for neutrophil elastase/lactoferrin were counted and the percentage of neutrophil elastase/lactoferrin positive cells was calculated.

### Video-Fluorescence Microscopy

Neutrophils were isolated as described in section Isolation of bovine PMNs. Again, 19,500 cells were seeded in a 12-well silicone chamber slide (Ibidi) and preincubated for 1 h at 37°C and 5% CO_2_. To detect DNA, neutrophils were incubated at 37°C for 20 min with Hoechst 33342 (final concentration 10 μg/mL, Sigma Aldrich) prior to a washing step with RPMI 1640. The medium was then exchanged once more and mucins (20 μg/μL) were added to the cells. Subsequently, cells were analyzed for 4 h using fluorescence microscopy (Carl Zeiss confocal laser scanning microscope LSM 800). Cells were monitored using a shooting interval of 1 picture/min.

### Hydrolysis of Purified Bovine Cervical Mucins

Isolated cervical mucins were hydrolyzed with 1 N acetic acid for 30 min at 80°C and 350 rpm in a thermos-shaker. Hydrolyzed samples were dried via lyophilization overnight, resuspended and dialyzed in water with Spectra-Por® Float-A-Lyzer® (G2 blue, 1 mL, MWCO 100 kDa, Sigma Aldrich) in order to remove mono-sialic acid. Successful hydrolysis was verified by lectin staining (please see section Lectin staining).

### C7 Modification of Bovine Cervical Mucins

The first step of the conversion of a 9-carbon sugar (sialic acid) to a C7-corpus was the oxidation via sodium-metaperiodate. Therefore, dried samples of isolated cervical mucins were resuspended in 50 μL of sodium acetate buffer (pH 5.5, 40 mM) and 4 μL of 0.25 M sodium-metaperiodate were added as described previously (33–35). Incubation, which took place for 3 h at 0°C without shaking was stopped by the addition of 10 μL of 3% ethylene-glycol (30 min, Rt). Subsequently, 64 μL of 0.2 N borohydride dissolved in 0.2 N sodium borate buffer pH 8.0 was added and incubated at 0°C overnight before samples were dried.

In order to determine the success of the oxidation of sialic acids with sodium-metaperiodate, sample aliquots were hydrolyzed with 0.2 N trifluoroacetic acid (TFA) for 4 h at 80°C. Dried samples as well as appropriate standards were then labeled with 1,2-diamino-4,5-methylenedioxybenzene (DMB) under the following conditions: 40 μL DMB reagent [9 mM sodium hydrosulfite, 0.5 M β-mercaptoethanol, 20 mM TFA, and 1.35 M DMB (Dojindo, Kumamoto, Japan)] and 40 μL of MilliQ water was added and incubated for 2 h, 55°C, 350 rpm and the labeling was stopped by the addition of 20 μL 0.2 M NaOH (36, 37). The separation was performed with LiCroCart 250-2 Merck and a SuperSpher 100 reverse-phase- C18 column as described previously (38, 39).

### Neuraminidase Treatment of Mucins

Samples were treated with an α-2,3;6;8 specific sialidase (New England Biolabs, Ipswich, Massachusetts, USA). The incubation of the α-2,3;6;8 neuraminidase (6 Units) in a final volume of 30 μL (1x Glyco buffer) took place for 5 min at 37°C. The reaction was stopped by heating the sample to 65°C for 10 min, followed by dialysis in water with Spectra-Por® Float-A-Lyzer® (G2 blue, 1 mL, MWCO 100 kDa, Sigma Aldrich) in order to remove mono-sialic acid.

### Lectin Staining

The exoglycosidase treatment and the chemical release of sialic acid residues were assessed by Western blotting agarose gels. A 0.8% agarose gel was prepared in 40 mM Tris-Acetate, 1 mM EDTA (TAE, pH 8.0) containing 0.1% sodium dodecyl sulfate (SDS). Samples for electrophoresis were dried and diluted 1 to 10 in sample buffer (1x TAE-buffer, 50% glycerol, 0.25% bromophenol blue, 1% SDS), according to Ramsey et al. (40). The gel was run for 90 min at 80 V. Then the gel was washed briefly with water, prior to incubating the gel in 4 × saline sodium citrate (SSC: 0.6 M sodium chloride, 60 mM trisodium citrate, pH 7.0), containing DTT (final concentration 10 mM) for 20 min at Rt. After a further washing step with DTT free 4 × SSC, the gel was placed onto a VacuGene XL blotting apparatus (GE Healthcare, Dornstadt, Germany) and samples were blotted onto a PVDF membrane under vacuum (60 mbar, 2 h) following the manufacturer's instructions. For lectin staining, membrane was then rinsed with PBS and blocked with 3% milk in PBS for 1 h. Lectins [Biotinylated Maackia Amurensis II (MAL II, final concentration 1 μg/mL, Vector, Burlingame, California, USA); Biotinylated Sambucus Nigra Lectin (SNA, final concentration 1 μg/mL, Vector)] were added and incubated for 30 min at Rt. Then further washing steps were carried out prior to application of HRP-coupled streptavidin (final concentration 50 mU/mL diluted in PBS-T, Roche). Incubation took place for 40 min at Rt, followed by 3 further washing steps. For visualization enhanced chemiluminescence reagent (ECL solution: 1.27 mM Luminol, 0.6 mM p- Coumaric acid, 0.008% H_2_O_2_) was used in combination with an imaging system (Bio-Rad). Experiments were performed in triplicate with freshly hydrolyzed or neuraminidase digested mucins.

### CellToxGreen Assay

Isolated PMNs were seeded in black 96-well plates at a total cell number of 150,000 cells/well. Cells were stimulated with 1.5 μM PMA and 3 μM ionomycin for 4 h. In addition, estrus mucins were added (final concentration 20 μg/μL) and co-incubated with 1.5 μM PMA and 3 μM ionomycin. Subsequently, CellToxGreen (Promega, Madison, Wisconsin, USA) was added as described in the user's manual. This assay is based on a cyanine dye that is not absorbed by viable cells, but exhibits an enhanced fluorescence signal, when binding to DNA occurs in cells with loss of their membrane integrity. Measurement of increasing fluorescence intensity was performed for 4 h every 15 min at 485 nm for excitation and 520 nm for emission. The received values were corrected by subtracting blank values and *t* = 0 h values, in order to determine the increase of the fluorescence signal.

### Sequence Alignments

Sequence alignments were performed using the EMBL EBI Clustal Omega tool (https://www.ebi.ac.uk/Tools/msa/clustalo/). Protein sequences used are available in the NCBI data bank (Accession numbers: human Siglec-8: XP_010813599.1; bovine Siglec-8: XP_010813599.1) The V-set Ig-like domain of human Siglec-8 was determined based on PDB 2n7b (https://www.ebi.ac.uk/pdbe/entry/pdb/2N7B). V-set Ig-like domain of bovine Siglec-8 was estimated by sequence alignment. The different colors indicate the properties of the amino acids. Red, small hydrophobic/aromatic amino acids; blue, acidic amino acids; magenta, basic amino acids; green: hydrophilic, polar and small amino acids.

### 3D Modeling

The modeling of the 3D structure of the binding-model between human as well as bovine Siglec-8 to 6′-S-sialyl-Lex was performed using UCSF Chimera 1.13.1. The structure of human Siglec-8 is based on that reported by Pröpster et al. (41) (PDB 2n7b). The structure of bovine Siglec-8 was generated using the Phyre^2^ web portal for protein modeling (42). The sequence of the V-set Ig like domain of bovine Siglec-8 is based on the performed alignment. The components of the glycans are differently labeled: sialic acid (purple), fucose (yellow), galactose-6-P (cyan), N-acetylgalactosamine (magenta), 3-aminopropan-1-ol (green).

### Statistical Analysis

Data sets were analyzed with Graph Pad Prism 7.0 software using paired ANOVA and a multiple-comparison Tukey test. The data have been initially evaluated by D'Agostino and Pearson test (*n* ≥ 8) or Shapiro-Wilk test (*n* < 8) to calculate the distribution of the values. The calculated differences were considered statistically significant at *p* ≤ 0.05. Statistically significant differences are given: ns, not significant; ^*^p ≤ 0.05; ^**^p ≤ 0.01; ^***^p ≤ 0.001; ^****^p ≤ 0.0001.

## Results

### Activating and Inhibitory Siglecs Are Expressed in Bovine PMNs

As mucins are very heavily glycosylated glycoproteins, carrying numerous sialylated glycans, an inhibitory effect on NETosis, induced by the interaction of bovine cervical mucins with Siglecs, is likely. To address this hypothesis, we firstly analyzed the range of Siglecs in isolated bovine PMNs by RT-qPCR.

In order to evaluate the isolation strategy, the amount of granular containing cells was calculated by measuring the side scattered light and the forward scattered light using a FACS system. Based on the obtained values, ~92% of the isolated cells show high internal granularity (Supplementary Figures 1A,B). In addition, neutrophil elastase and lactoferrin were visualized. Approximately 96 and 98% of the analyzed cells were lactoferrin and neutrophil elastase positive, respectively (Supplementary Figures 1B,C). Thus, it can be assumed that mainly PMNs were isolated. However, low numbers of other blood cells, such as eosinophils and lymphocytes, might be present.

**Figure 1:**
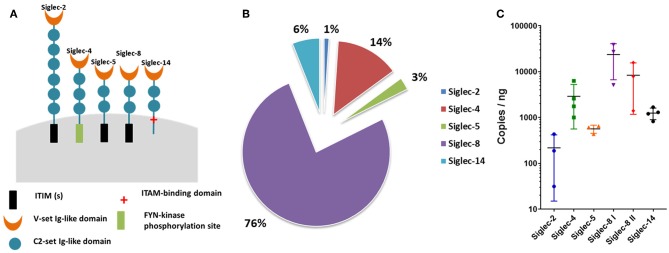
Siglecs expressed in bovine neutrophils. **(A)** Cartoon of the Siglecs expressed by bovine neutrophils and their functional intracellular domains. The number of C2-set Ig-like domains was determined by SMART (http://smart.embl-heidelberg.de/smart/set_mode.cgi?NORMAL=1) based on the following NCBI sequences: Siglec-2: XP_024834447.1; Siglec-4: XP_0248341235.1; Siglec-5: XP_005219620.2; Siglec-8: XP_010813599.1; Siglec-14: XP_019834314.1. **(B)** Pie chart of the percentage Siglec expression in bovine neutrophils. qPCR raw data was normalized against the reference genes. Siglec-2, -4, -5, -8, and -14 were detected by RT-qPCR and the total of the detected Siglecs was set to 100%. **(C)** Determined copies of Siglecs per ng RNA used during qPCR. X-axis shows the Siglecs expressed by bovine neutrophils; Y-axis shows the log 10 values. Mean values and standard deviations are displayed in the diagrams (Siglec-2, Siglec-5, Siglec-8 I, Siglec-8 II, *n* = 3 different animals; Siglec-4, Siglec-14, *n* = 4 different animals). For Siglec-8, values generated with two different primer pairs are displayed.

The RT-qPCR analyses demonstrated that mRNA products for Siglec-2, -4, -5, -8, and -14 were detectable in the isolated cell populations (Figure 1). Since the highest copy numbers were detectable for Siglec-8 (Figures 1B,C) and in humans this inhibitory Siglec is only present on eosinophil/mast cells, but absent from neutrophils, we derived two different sets of primers. The primers pairs are specific for different regions of the bovine Siglec-8 cDNA sequence to verify the qPCR results. In addition, the resulting PCR products were sequenced. All results confirmed Siglec-8, as the Siglec with the highest copy number in the isolated cell populations (Figure 1B). The second largest Siglec fraction was Siglec-4, also known as myelin-associated glycoprotein (MAG) (Figures 1B,C). Finally, transcripts were detected for Siglec-5 and Siglec-2, which function as inhibitory receptors.

**Figure 2:**
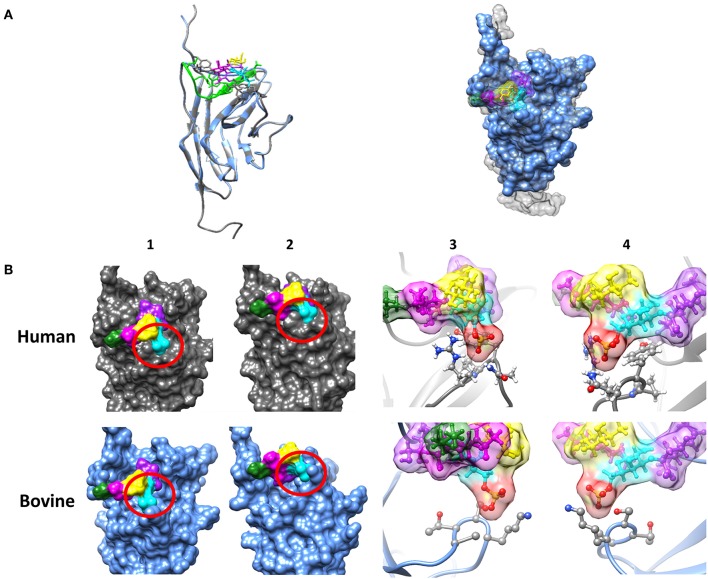
Investigation of the sialic acid binding properties of human and bovine Siglec-8. **(A)** Overlay of the tertiary structure of hSiglec-8 (gray) and bovine Siglec-8 (blue). The components of the glycan are differently labeled: sialic acid (purple), fucose (yellow), galactose-6-P (cyan), N-acetylgalactosamine (magenta), 3-aminopropan-1-ol (green). **(B)** Differences in the tertiary structure of hSiglec-8 (gray) and bovine Siglec-8 (blue). Different views of the glycan-binding domain are shown (1–4). (3,4) 6-P is labeled in red. All figures were created by Chimera 1.13.1. Red circle demonstrates potential differences in glycan-binding properties. The structure of hSiglec-8 is based on Pröpster et al. (41) and the structure of bovine Siglec-8 was designed using Phyre^2^.

**Figure 3:**
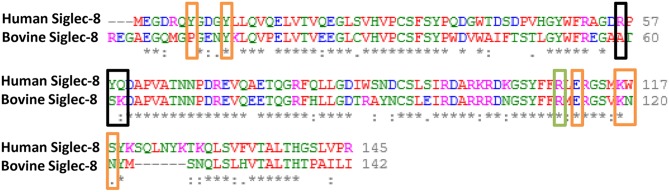
Comparison of the sialic acid binding domain between human and bovine Siglec-8. Sequence alignment of the V-set domain of hSiglec-8 (pdb: 2N7B) and the predicted sialic acid binding domain of bovine Siglec-8 (NCBI: XP_010813599.1). Sequence alignments were performed using the Clustal Omega tool of EMBL-EBI. The different colors indicate the properties of the amino acids. Red, small hydrophobic/aromatic amino acids; blue, acidic amino acids; magenta, basic amino acids; green, hydrophilic, polar and small amino acids. The boxes indicate amino acids, which are mainly involved in binding of the 6′-S-sialyl-Lex motif based on Pröpster et al. (41): black boxes indicate amino acids required for the binding of galactose-6-S. Orange boxes indicate amino acids mainly involved in Neu5Ac binding. In addition, the highly conserved Arginine (R), that is essential for sialic acid binding, is boxed in green.

Siglec-8 has not only the highest copy numbers but also the highest circumscribed ligand preference of the detected Siglecs [based on human Siglec data (43, 44)]. In contrast to hSiglec-8, which prevalently recognizes a very special glycan motif, namely 6′-sulfo-sialyl-Lewis X (6′-S-sialyl-Le^x^), Siglec-2, -4, -5, and -14 can bind several sialylated motifs, which are frequently present on numerous sialylated glycans (44). For this reason, the glycan-binding domain of hSiglec-8 was compared with the presumed binding domain of bovine Siglec-8 (Figure 2). For human Siglec-8 the selective binding mechanism for the sulfated galactose residue of the 6′-S-sialyl-Le^x^ motif is well-known (41). The amino acids R56 and Q59 stabilize the binding with the negatively charged sulfate group of the 6′-S-galactose (6′-S-Gal) residue. In addition, Y58 interacts with the galactopyranose ring. The overlay of the human and the calculated bovine 3D structure suggests that the binding area for 6′-S-Gal is missing in bovine Siglec-8 (Figure 2B). A sequence alignment of the glycan-binding domain of hSiglec8 (pdb: 2N7B) and the presumed binding domain of bovine Siglec-8 (XP_010813599.1) confirmed the calculated model. The amino acid R56 is replaced by A, Y58 is replaced by S and Q59 is replaced by K. Consequently, all amino acids, which are essential for the circumscribed ligand preference to 6′-S-sialyl-Le^x^, are absent in bovine Siglec-8 (Figure 3). In contrast, most amino acids responsible for sialic acid binding are highly conserved in human and bovine Siglec-8. Thus, remarkable structural differences exist mainly in the area that is essential to interact with the circumscribed 6′-S-Gal residue.

In summary, the results demonstrated that prevalently inhibitory Siglecs were expressed in bovine isolated cell population and, as determined by copy numbers, Siglec-8 was the most highly expressed.

### Cervical **Mucins** Inhibit PMA/Ionomycin and LPS Induced NET Formation

**Figure 4:**
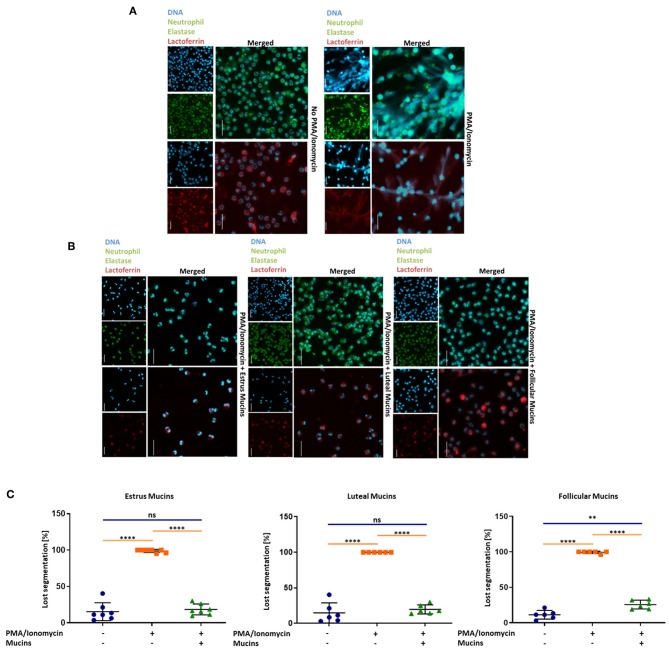
Twenty microgram/microliter cervical mucins prevent NET release induced by a combination of 1.5 μM PMA and 3 μM ionomycin. **(A)** DNA fluorescence staining (DAPI) and staining of neutrophil elastase (Green) and lactoferrin (Red) of bovine neutrophils without any stimulation and stimulated with PMA/ionomycin. The term “merged” indicates the overlay of the nuclei staining with the staining of neutrophil elastase or lactoferrin, respectively. Scale bars: 20 μm. **(B)** DNA fluorescence staining and neutrophil elastase (Green) and lactoferrin (Red) of bovine neutrophils stimulated with PMA/ionomycin co-incubated with 20 μg/μL bovine cervical mucins from different stages of the estrus cycle. The term “merged” indicates the overlay of the nuclei staining with the staining of neutrophil elastase or lactoferrin, respectively. Scale bars: 20 μm. **(C)** The percentage of activated cells after 4 h of stimulation, without any stimulation, stimulated with PMA/ionomycin and stimulated with PMA/ionomycin co-incubated with 20 μg/μL bovine cervical mucins from different stages of the estrous cycle. Total cell number and cells with remaining segmented nuclei were counted and the percentage of activated neutrophils was calculated. Mean values and standard deviations are displayed in the diagrams (estrus mucins *n* = 7 different animals; luteal and follicular mucins *n* = 6 different animals). Paired ANOVA and a multiple-comparison Tukey test were applied. Statistically significant differences are given: ns, not significant; **p ≤ 0.01; ****p ≤ 0.0001. For higher magnification of the stained neutrophils please see Supplementary Figure 2.

Since the qPCR data revealed that mainly inhibitory Siglecs are expressed in bovine neutrophils, we were interested in the potential of sialylated cervical mucins to inhibit NET release. To this end, we stimulated bovine neutrophils in the presence and absence of cervical mucins. The mucins were applied with a final concentration of 20 μg/μL, since mucins are mainly present in concentrations between 10 and 50 μg/μL in mucus (45). As shown in Figure 4, unstimulated neutrophils exhibit a characteristic segmented nucleus. When neutrophils are stimulated with a combination of 1.5 μM PMA and 3 μM ionomycin nearly all nuclei lose their segmented structure and NET is released (Figure 4 and Supplementary Figure 2). In addition to DNA, neutrophil elastase and lactoferrin were visualized by immunofluorescence staining. Both proteins are located in selected granules of PMNs and during NETosis the content of these granules are combined with the meshwork of DNA (5, 6, 46–48). The obtained results demonstrate that the released chromatin is decorated with neutrophil elastase and lactoferrin (Figure 4). However, when cervical mucins from different phases of the estrous cycle were applied, the activation of neutrophils seems to be inhibited (Figures 4A,B). The nuclei still show segmented structure similar to unstimulated neutrophils and the immunostaining of lactoferrin and neutrophil elastase exhibited the maintenance of the internal granularity. In addition to stimulated PMNs, cervical mucins were added to un-stimulated cells and no alterations were detectable (Supplementary Figure 3, **Video**).

Since NETosis comes along with membrane rupture, we further analyzed membrane integrity. Samples of estrus mucin were applied to stimulated neutrophils and membrane integrity was determined using CellToxGreen Assay. After 4 h of neutrophil stimulation with PMA/ionomycin, the fluorescence intensity increased, due to the initiated NETosis (Supplementary Figure 4). However, when cervical estrus mucins were applied, the fluorescence signal remained unchanged indicating that membrane integrity is maintained. Comparable fluorescence intensities to those of unstimulated neutrophils were achieved. In order to test the efficiency of mucins to inhibit the activation of PMNs by PMA/ionomycin, different concentrations of cervical mucins were applied. Whereas, 1 μg/μL mucin or 5 μg/μL mucin showed no significant effects on NET formation induced by the combination of PMA and ionomycin, the addition of 10 μg/μL mucin reduced the release of NET (Supplementary Figure 5) by ~40%. Thus, with increasing concentrations of mucins, fewer numbers of PMNs were activated demonstrating a concentration dependent mechanism.

**Figure 5:**
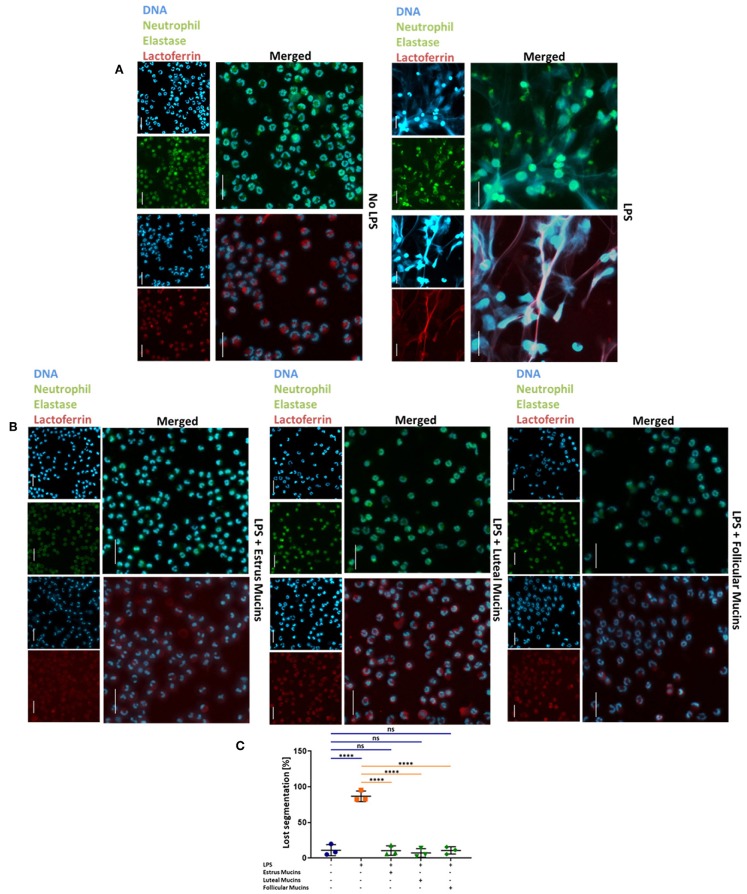
Cervical mucins prevent NET release induced by 20 μg/mL LPS. **(A)** DNA fluorescence staining (DAPI) and staining of neutrophil elastase (Green) and lactoferrin (Red) of bovine neutrophils without any stimulation and stimulated with LPS from *pseudomonas aeruginosa*. The term “merged” indicates the overlay of the nuclei staining with the staining of neutrophil elastase or lactoferrin, respectively. Scale bars: 20 μm. **(B)** DNA fluorescence staining (DAPI) and staining of neutrophil elastase (Green) and lactoferrin (Red) of bovine neutrophils stimulated with 20 μg/mL LPS co-incubated with 20 μg/μL bovine cervical mucins. The term “merged” indicates the overlay of the nuclei staining with the staining of neutrophil elastase or lactoferrin, respectively. Scale bars: 20 μm. **(C)** The percentage of activated cells after 4 h of stimulation, without any stimulation, stimulated with LPS and stimulated with LPS co-incubated with 20 μg/μL bovine cervical mucins from different stages of the estrus cycle. Total cell number and cells with remaining segmented nuclei were counted and the percentage of activated neutrophils was calculated. Mean values and standard deviations are displayed in the diagrams (*n* = 3 independent isolations from 2 different animals). Paired ANOVA and a multiple-comparison Tukey test were applied. Statistically significant differences are given: ns, not significant; ****p ≤ 0.0001. For higher magnification of the stained neutrophils please see Supplementary Figure 2.

In addition to the combination of PMA and ionomycin, 20 μg/mL LPS was used as NET inducer. According to Pieterse and colleagues LPS from *Pseudomonas aeruginosa* is a potent stimulator for NETosis (49). Comparable to the PMA/ionomycin stimulation, neutrophils undergo NETosis in the presence of LPS (Figure 5A). Again, the DNA filaments are highly decorated with the granular proteins neutrophil elastase and lactoferrin. When cervical mucins are present during stimulation, the effects of LPS was abolished (Figure 5B). Independent of the stage of estrous cycle NETosis was prevented by mucins and neutrophils retained their segmented nuclei (Figure 5C). However, in contrast to PMA/ionomycin, mucins at concentrations of 1 μg/μL completely inhibited LPS induced NETosis (Supplementary Figure 6).

### The Inhibition of NET Formation Depends on the Sialylation Status of Cervical Mucins

**Figure 6:**
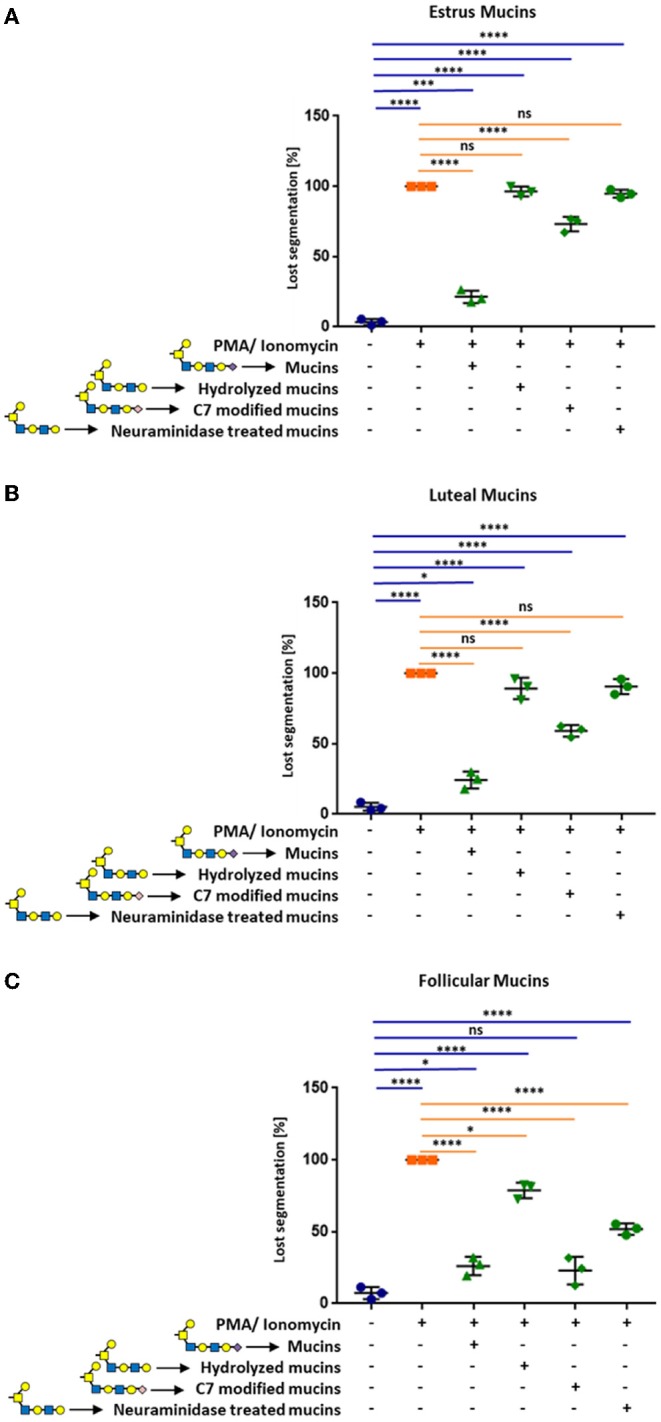
Cervical mucins inhibit the release of NETs induced by 1.5 μM PMA in combination with 3 μM ionomycin by sialic acid on its surface. The experiments were performed with mucins from **(A)** estrus, **(B)** luteal, and **(C)** follicular samples. Untreated mucins, as well as hydrolyzed, C7 modified and neuraminidase treated mucins were applied to stimulated neutrophils in a final concentration of 20 μg/μL. The percentage of activated cells was calculated by counting the segmented nuclei as well as the total cell number. Mean values and standard deviations are displayed in the diagrams (*n* = 3 different animals). Paired ANOVA and a multiple-comparison Tukey test were applied. Statistically significant differences are given: ns, not significant, *p ≤ 0.05; ***p ≤ 0.001; ****p ≤ 0.0001. Glycan illustration: Yellow square: N-acetylgalactosamine, Blue square: N-acetylglucosamine, Yellow circle: Galactose, Purple diamond: Sialic acid, rose diamond: C7 modified sialic acid.

To investigate, whether sialic acids are involved in the observed inhibition of NET formation, we first released sialic acid residues from bovine cervical mucins using acidic conditions. The released sialic acid residues were removed by dialysis. The mild hydrolysis led to a significant decrease of the sialylation status, as verified by lectin staining of agarose gels (Supplementary Figure 7). When we applied the acidic treated estrus mucins, no inhibitory activity against the stimulation of neutrophils was observed and NET formation occurred (Figure 6A and Supplementary Figure 8). Similar results were achieved when desialylated follicular mucins were used (Figure 6B and Supplementary Figure 9). However, unlike follicular and estrus mucins, where released NET fibers were visible after desialylation, the reduced sialylation of luteal mucins lead to an activation of neutrophils and a loss of the segmented nuclei structure but NET release was prevented (Figure 6C and Supplementary Figure 10).

Since the hydrolytic conditions may also result in additional alteration of the mucin structure, we mildly oxidized the terminal sialic acid residues. The oxidation of sialic acids with sodium periodate is a well-established method that specifically modifies the exocyclic side chain of unmodified terminal sialic acid residues. During oxidation, C9 and C8 are released resulting in a C7-backbone. Subsequently, the resulting aldehyde group is reduced and a hydroxyl group is formed at C7 of the terminal sialic acid residues (34). This chemical reaction was confirmed by HPLC (Supplementary Figure 11). The conversion of the C9-carbon sugar into a C7-carbon sugar, partially inhibited the observed effects of estrus and luteal mucins (Figure 6 and Supplementary Figures 8–10). In contrast to oxidized luteal and estrus mucins, the C9/C7 conversion of follicular mucins has no effect (Figure 6C and Supplementary Figure 9). Oxidized and native follicular mucins showed comparable effects.

In addition, neuraminidase was employed to enzymatically release sialic acid residues. After digestion, neuraminidase was heat-inactivated and released sialic acids were removed by dialysis. The enzymatic treatment completely eliminated the previously detected inhibitory effects of estrus and luteal mucins (Figures 6A,C). However, in the case of follicular mucins ~50% of the neutrophils were still inactive (Figure 6B). This might be the consequences of a poor detachment of release of sialic acid by neuraminidases. The staining with SNA demonstrated that the detachment was not efficient for α2,6-linked sialic acid in the case of follicular mucins (Supplementary Figure 12).

In summary, the results demonstrate that cervical mucins from different phases of the estrous cycle have the capability to suppress the activation of neutrophils to form NET in a sialic acid dependent manner.

## Discussion

The route for sperm to reach the oviduct is challenging. They have to evade areas with low pH, to pass through highly viscous areas and to navigate or circumvent the female immune system (50, 51). After mating, neutrophils are recruited in cervical, uterine and fallopian tube tissue to protect the female reproductive tract against bacterial contamination (52). In mated Fresian cows, for instance, 6.4 ×10^5^ PMNs were counted in the lumen of the cervix (52). The polymorphonuclear cells do not just target pathogens present in semen but also spermatozoa (3, 50). Most bacteria and spermatozoa are eliminated via phagocytosis and probably degranulation of antimicrobial peptides/proteins in addition to the release of reactive oxygen species (ROS) additionally damage spermatozoa. For example, it is known that ROS can influence the motility and the survival of sperms (53–55). However, besides these mechanisms, the formation of extracellular traps appear to play a role in reproductive immunology and the tight control of PMN response is critical to normal fertility (2). The mechanisms of PMN regulation that control pathogen phagocytosis or NET entrapment are poorly understood (56). The same applies for the possibility of phagocytosis triggered NETosis. Interestingly, ROS is a key player during phagocytosis (57, 58) and NETosis (6). However, during phagocytosis ROS is mainly produced in the phagosomal lumen, whereas during NETosis cytosolic ROS production is induced (56). Remarkably, PMNs, which have phagocytosed apoptotic cells, seems to be unable to form NET (59). However, it is unknown, if phagocytosed sperm induce similar effects inhibiting an exaggerated NET formation to prevent a blockade of the cervical canal by NET and thus, to ensure a transit of spermatozoa through this duct system.

While this would be one possibility to silence the induction of NETosis, we hypothesized that sialylated cervical mucins are further modulators to influence the activation of neutrophils, since sialylated glycans on endogenous cells as well as pathogens have been shown to inhibit the formation of NET (12, 60–62). In the cervix, neutrophils and sperm have to penetrate the viscous mucus that consists mostly of mucins, glycoproteins that are frequently decorated with sialic acid residues. For this reason, we have investigated the inhibitory capabilities of bovine cervical mucins to modulate the activation of neutrophils.

In a first set of experiments, bovine PMNs were isolated and tested for the expression of Siglecs. Surprisingly, an inhibitory Siglec, that was assigned as Siglec-8 in cattle, is mainly expressed in the isolated cell fractions. In humans, Siglec-8 is assumed to be a mast cell/eosinophil specific receptor preferring the binding of 6′-S-sialyl-Le^x^ (41, 44, 63, 64). Sequence alignments and structure comparison between the glycan-binding domain of hSiglec-8 and the assumed binding domain of bovine Siglec-8 exhibited structural differences suggesting that these changes are associated with alterations in glycan-binding properties and a loss of its binding preference for the 6′-S-Gal motif. Besides Siglec-8, Siglec-4 represents a further Siglec, which is commonly not expressed in neutrophils in humans. In contrast to Siglec-8, Siglec-4 does not contain ITIMs or ITIM-like sequences but a FYN kinase phosphorylation site in cattle (65, 66) and is predominantly known to be expressed in Schwann cells (67), as well as oligodendrocytes, stabilizing myelin-axon interactions (43). In contrast to the low copy numbers of the B-cell marker Siglec-2, which might be a contamination from lymphocytes, it is unlikely that the high mRNA values for Siglec-4 and Siglec-8 are from contaminating cells. More than 95% of the isolated cells show the characteristic lactoferrin and neutrophils elastase staining of PMNs, whereby neutrophil elastase is a marker for azurophilic granules in PMNs, lactoferrin is stored in specific granules (46, 47). However, it is impossible to fully exclude this possibility. Nevertheless, it is clear that prevalently inhibitory Siglecs were expressed in the isolated cell fractions.

To investigate the potential of cervical mucins to inhibit NET release, cervical mucins of different phases of the estrous cycle were isolated and applied to neutrophils, which were stimulated with LPS or PMA/ionomycin. The obtained results showed that not only estrus but also luteal and follicular mucins efficiently inhibited the formation of NET independent of the applied stimulus for NETosis. Remarkably, the chemical modification and release of the sialic acid residues on cervical mucins decrease the inhibitory capability. Therefore, a Siglec-dependent inhibition of NET release is more likely.

However, cervical mucins are not the only known immuno-modulatory biomolecules in the female reproductive tract after mating. If NET is formed, DNases in the seminal plasma are able to degrade the released DNA framework and therefore, protect sperm from entrapment (1). Interestingly, it seems that a loss of DNase activity is associated with subfertility in the stallion (1). Nevertheless, DNases can only protect spermatozoa within a limited radius from the site of ejaculate deposition (the vagina). The same applies for sialic acid polymers in ejaculates, which can neutralize in a chain length dependent manner the cytotoxic characteristic of histones, without a neutralization of the anti-microbial capacity of lysine-rich histones (68, 69). Even though, few spermatozoa are polysialic acid positive (70), most of these protective sugar units against NET will get lost during the cervical transit. However, during the passage through the cervix, sperm penetrates a dense meshwork of sialylated mucins secreted by the cervical epithelium. Thus, as they come in contact with neutrophils in this area, we propose that mucin-associated sialic acid residues prevent an exaggerated NET formation. Similar to sialylated structures on circulatory erythrocytes, cervical mucins may counteract in a sialic acid dependent manner an over-active neutrophil NET response so that more sperms can continue their journey toward the site of fertilization.

In addition to a possible regulatory effect of sialic acid residues on mucins, sialic acid residues are also evident on the spermatozoa glycocalyx. These play a role in protection against phagocytosis and sperm desialylation results in a loss of these protective effects (12). Thus, two powerful killing mechanisms of neutrophils (phagocytosis and NET) might be modulated in a sialic acid dependent manner by both the spermatozoan itself as well as cervical mucins to ensure the survival of sufficient numbers of spermatozoa in the female reproductive tract for the fertilization of the ovum.

In sum, the results demonstrated that cervical mucins inhibit the formation of NET. The inhibitory capacity strongly depends on the sialylation status of the mucins demonstrating once more the central role of these acidic sugar residues in reproductive biology (71–74). Since mucins are generated by epithelial cells throughout the body, comparable mucin dependent mechanisms may also play a role in other organs, such as the lung or the gastrointestinal tract. Thus, mucins as a natural and bio-degradable product might be a further target to develop novel clinical applications for the modulation of NETosis.

## Data Availability Statement

The used datasets of Siglec 8 for this study were obtained from NCBI data bank (https://www.ncbi.nlm.nih.gov/). The datasets for the 3D-model of human Siglec-8 were obtained from Pröpster et al. (41) (PDB 2n7b). All novel datasets generated for this study are included in the manuscript Supplementary Files.

## Ethics Statement

Ethical review and approval was not required for the animal study because bovine cervices were only collected from animals sent to the abattoir for meat production. Bovine neutrophils were isolated from blood samples received from the slaughterhouse or from leftover blood samples of regulatory blood collections from other projects (7221.3-1-010/16-1). Both the slaughter processes and the blood sampling were performed in accordance with the guidelines, applicable laws and provisions for ethical regulations.

## Author Contributions

KB, AR, MG, TV, and KZ performed the experiments and analyzed in addition to CR and SG the data. The manuscript was written through contributions of all authors. All authors have given approval to the final version of the manuscript.

### Conflict of Interest

The authors declare that the research was conducted in the absence of any commercial or financial relationships that could be construed as a potential conflict of interest.

## Abbreviations

DAPI: 4′,6-Diamidin-2-phenylindol
DMB: 1,2-diamino-4,5-methylenedioxybenzene
DTT: Dithiothreitol
EDTA: Ethylenediaminetetraacetic acid
HRP: Horse-rabbit-peroxidase
ITIM: Immunoreceptor tyrosine-based inhibition motif
ITAM: Immunoreceptor tyrosine-based activation motif
Gal-6′-S′: 6-S′Galactose
6′-S-sialyl Le^x^: 6′sulfo-sialyl-lewis X
Gdn HCl: Guadinium hydrochloride
LPS: Lipopolysaccharides
GlcNAc: N-acetylgalactosamine
MAG: Myelin-associated glycoprotein
MAL II: Maackia Amurensis II
NETs: Neutrophil extracellular traps
PFA: Paraformaldehyde
PCR: Polymerase chain reaction
PenStrep: Penicillin/streptavidin
PMA: Phorbol myristate acetate
PBS: Phosphate buffered saline
PMNs: Polymorphonuclear neutrophils
RT-qPCR: Real-Time quantitative Polymerase Chain Reaction
PVDF: Polyvinylidine fluoride
Rt: Room temperature
ROS: Reactive oxygen species
SDS: Sodium dodecyl sulfate
Siglec: Sialic acid-binding immunoglobulin-like lectins
SNA: Sambucus Nigra Lectin
SSC: saline sodium citrate
TFA: Trifluoroacetic acid
TAE buffer: TRIS-acetate buffer.
