# Supplementary material for: Sialylated Cervical Mucins Inhibit the Activation of Neutrophils to Form Neutrophil Extracellular Traps in Bovine in vitro Model
Source: Front Immunol. 2019 Nov 6;10:2478. doi: 10.3389/fimmu.2019.02478 (PMC6851059; doi:10.3389/fimmu.2019.02478)
Supplement: Supplementary file 1 [file Data_Sheet_1.zip › Figures/Figure 1.pdf]

Supplementary Material

1 Supplementary Figures

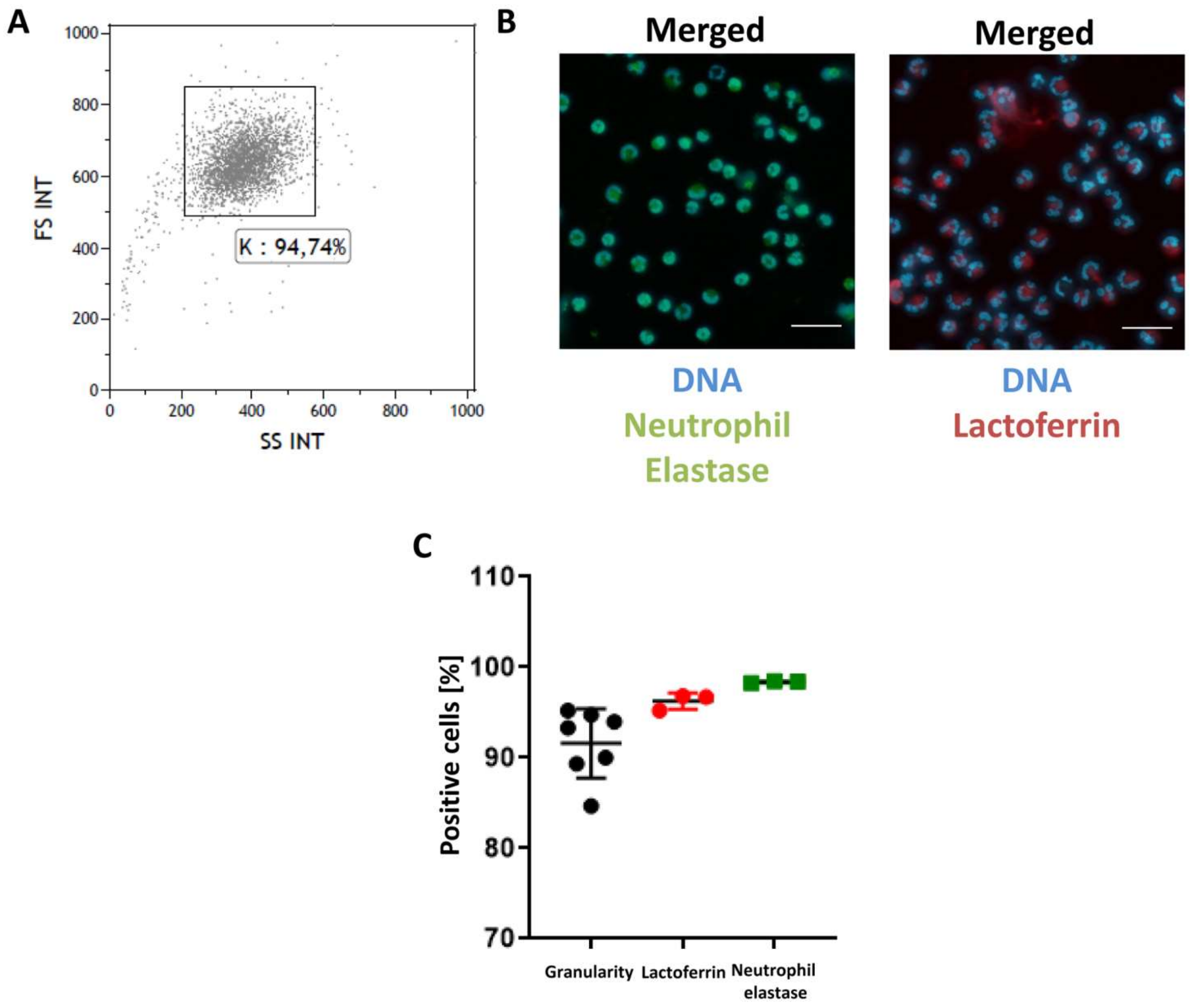

Supplementary Figure 1. Characterization of the isolated cell population. A) Granularity and size of isolated blood cells was examined by measuring the side scattered light (SSC) and the forward scattered light (FSC) as shown by the exemplary FACS plot. B) In addition, the ratios of neutrophil elastase/ lactoferrin positive cells were determined. Experiments were performed 3 times and each 3 randomly taken pictures were analyzed. In addition, a nucleus staining was performed using DAPI. The term “merged” indicates the overlay of the nuclei staining (Blue) with the staining of neutrophil elastase (Green) or lactoferrin (Red), respectively. C) The mean values of the calculated ratio of positive cells and standard deviations are shown for cell with high granularity (n = 7 different animals) in addition to lactoferrin (n = 3 independent isolations from 2 different animals) and neutrophil elastase (n = 3 independent isolations from 2 different animals) positive cells.
