# Supplementary material for: Sialylated Cervical Mucins Inhibit the Activation of Neutrophils to Form Neutrophil Extracellular Traps in Bovine in vitro Model
Source: Front Immunol. 2019 Nov 6;10:2478. doi: 10.3389/fimmu.2019.02478 (PMC6851059; doi:10.3389/fimmu.2019.02478)
Supplement: Supplementary file 1 [file Data_Sheet_1.zip › Figures/Figure 11.pdf]

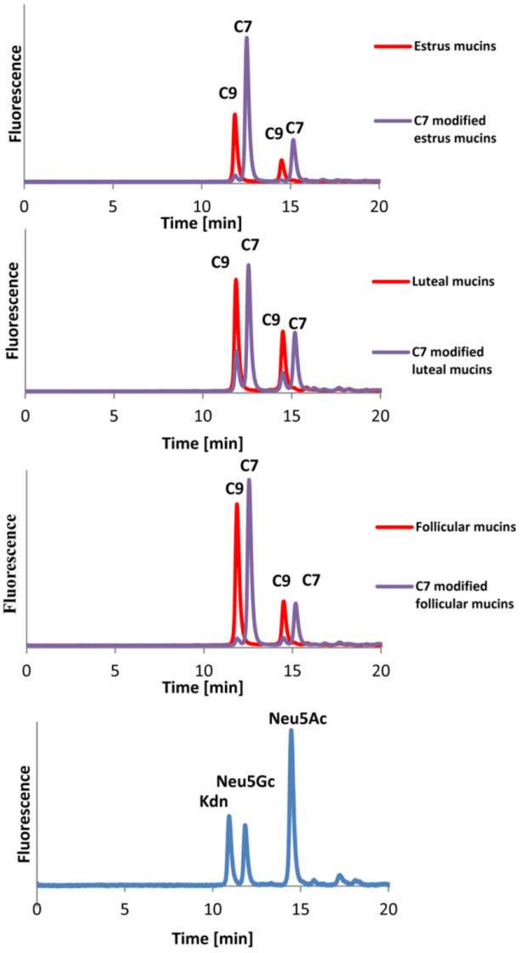

Supplementary Figure 11. Validation of the C9/C7 application of bovine cervical mucins. Red lines show non treated mucins, whereas the purple lines show C7 modified mucins. The blue chromatogramm displays the used standard. Three independent experiments were performed.
