# Supplementary material for: Sialylated Cervical Mucins Inhibit the Activation of Neutrophils to Form Neutrophil Extracellular Traps in Bovine in vitro Model
Source: Front Immunol. 2019 Nov 6;10:2478. doi: 10.3389/fimmu.2019.02478 (PMC6851059; doi:10.3389/fimmu.2019.02478)
Supplement: Supplementary file 1 [file Data_Sheet_1.zip › Figures/Figure 12.pdf]

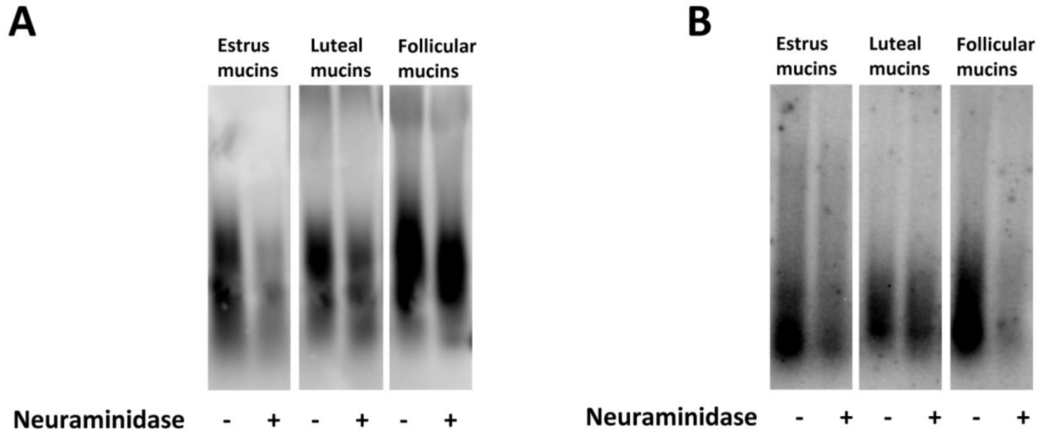

Supplementary Figure 12. Neuraminidase digestion of bovine cervical mucins. In order to confirm neuraminidase digestion we performed agarose gels and stained blotted PVDF membranes with the lectins A) SNA and B) MAL II. Each three independent experiments were performed.

.
