# Supplementary material for: Sialylated Cervical Mucins Inhibit the Activation of Neutrophils to Form Neutrophil Extracellular Traps in Bovine in vitro Model
Source: Front Immunol. 2019 Nov 6;10:2478. doi: 10.3389/fimmu.2019.02478 (PMC6851059; doi:10.3389/fimmu.2019.02478)
Supplement: Supplementary file 1 [file Data_Sheet_1.zip › Figures/Figure 2.pdf]

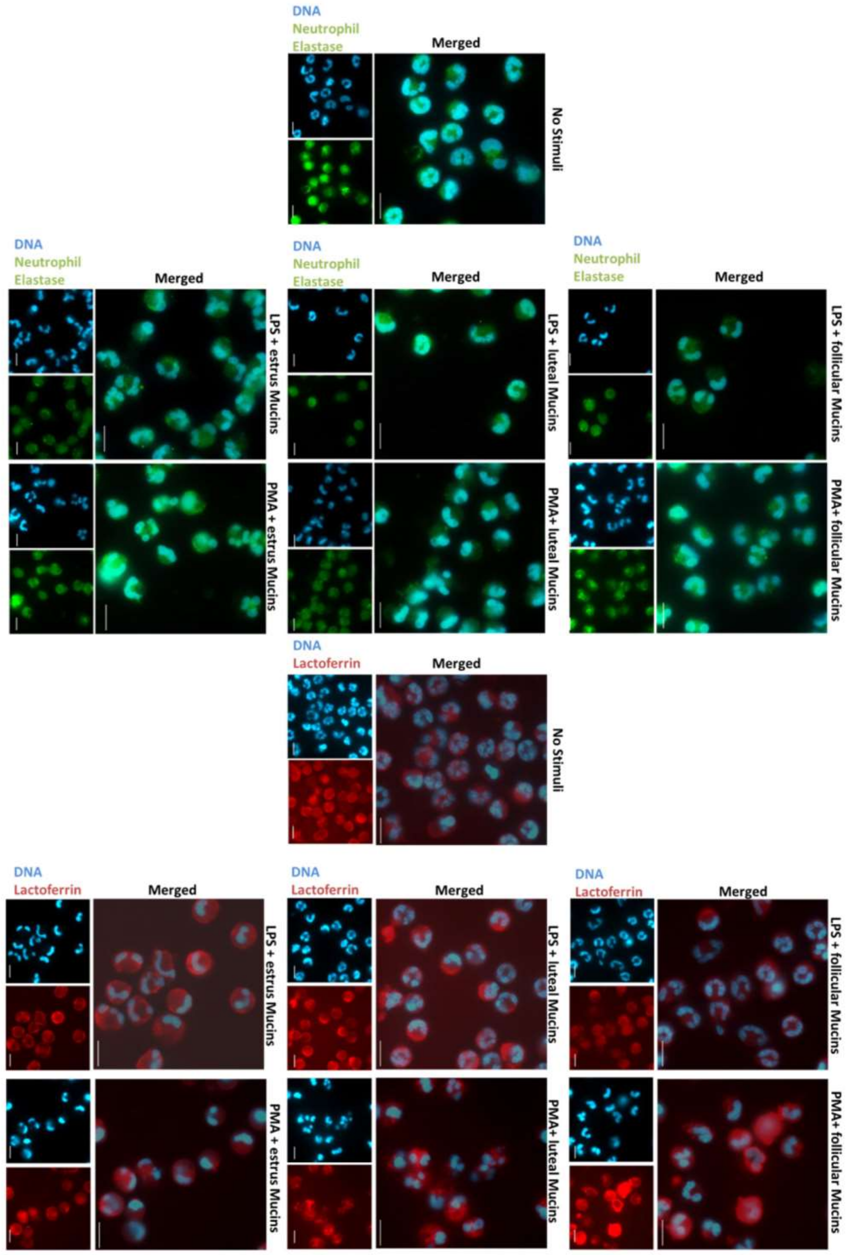

Supplementary Figure 2. Nuclei retain their segmented structure independently of the used stimuli when mucins were applied. A) DNA fluorescence staining (DAPI) and staining of neutrophil elastase (Green) of bovine neutrophils without any stimulation and stimulated with 20 µg/mL LPS from *pseudomonas aeruginosa* or a combination of PMA (1.5 µM) and ionomycin (3 µM) co-incubated with 20 µg/µL bovine cervical mucins of different stages of estrous cycle. The term "merged" indicates the overlay of the nuclei staining with the staining of neutrophil elastase. Scale bars: 10 µm. B) DNA fluorescence staining (DAPI) and staining of lactoferrin (Red) of bovine neutrophils without any stimulation and stimulated with 20 µg/mL LPS from *pseudomonas aeruginosa* or a combination

of PMA (1.5 μM) and ionomycin (3 μM) co-incubated with bovine cervical mucins of different stages of estrous cycle. The term “merged” indicates the overlay of the nuclei staining with the staining of lactoferrin. Scale bars: 10 μm.
