# Supplementary material for: Sialylated Cervical Mucins Inhibit the Activation of Neutrophils to Form Neutrophil Extracellular Traps in Bovine in vitro Model
Source: Front Immunol. 2019 Nov 6;10:2478. doi: 10.3389/fimmu.2019.02478 (PMC6851059; doi:10.3389/fimmu.2019.02478)
Supplement: Supplementary file 1 [file Data_Sheet_1.zip › Figures/Figure 3.pptx]

## Slide 1
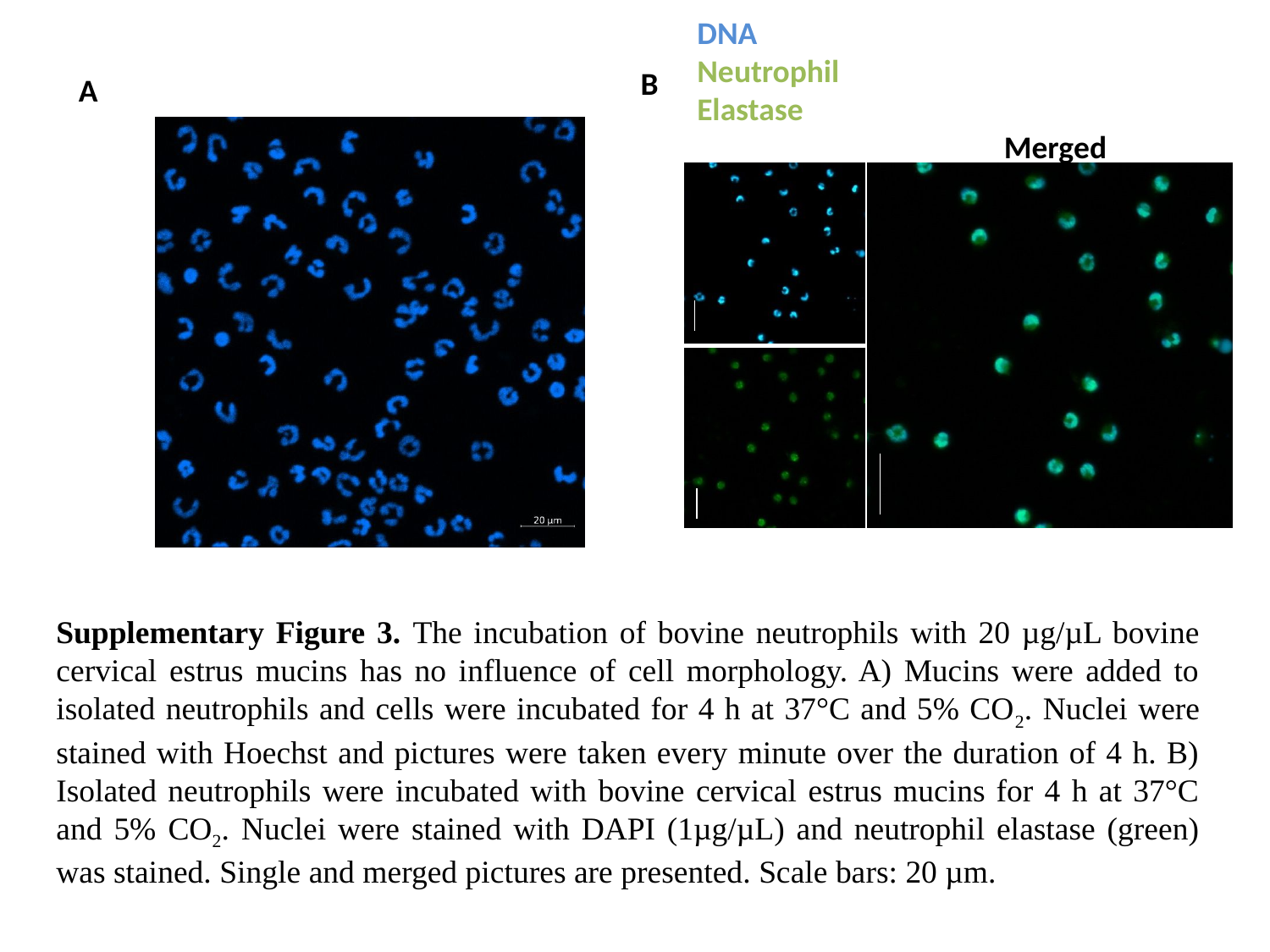

DNA
Neutrophil Elastase
B
A
Merged
Supplementary Figure 3. The incubation of bovine neutrophils with 20 µg/µL bovine cervical estrus mucins has no influence of cell morphology. A) Mucins were added to isolated neutrophils and cells were incubated for 4 h at 37°C and 5% CO2. Nuclei were stained with Hoechst and pictures were taken every minute over the duration of 4 h. B) Isolated neutrophils were incubated with bovine cervical estrus mucins for 4 h at 37°C and 5% CO2. Nuclei were stained with DAPI (1µg/µL) and neutrophil elastase (green) was stained. Single and merged pictures are presented. Scale bars: 20 µm.
