# Supplementary material for: Sialylated Cervical Mucins Inhibit the Activation of Neutrophils to Form Neutrophil Extracellular Traps in Bovine in vitro Model
Source: Front Immunol. 2019 Nov 6;10:2478. doi: 10.3389/fimmu.2019.02478 (PMC6851059; doi:10.3389/fimmu.2019.02478)
Supplement: Supplementary file 1 [file Data_Sheet_1.zip › Figures/Figure 4.pdf]

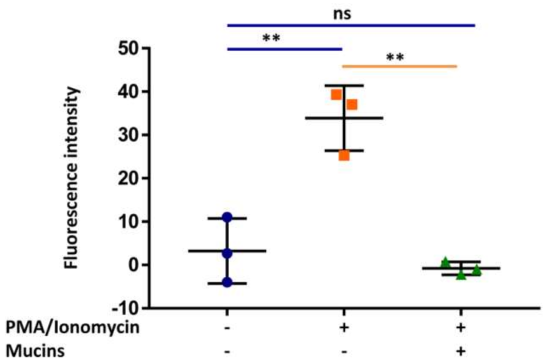

Supplementary Figure 4. Mucins prevent the perforation of the membrane. Bovine neutrophils were incubated with and without PMA/ionomycin and in addition, 20 µg/µL mucins were added and co-incubated with 1.5 µM PMA and 3 µM ionomycin. Measurement of increasing fluorescence intensity was performed for 4 h at 485 nm for excitation and 520 nm for emission. Values were calculated by subtracting blank values and t=0 h values, in order to determine the increase of the fluorescence signal. Mean values and standard deviations are displayed in the diagrams (n = 3 different animals). Paired ANOVA and a multiple-comparison Tukey test were applied. Statistically significant differences are given: ns, not significant; $^{**}p \leq 0.01$.
