# Supplementary material for: Sialylated Cervical Mucins Inhibit the Activation of Neutrophils to Form Neutrophil Extracellular Traps in Bovine in vitro Model
Source: Front Immunol. 2019 Nov 6;10:2478. doi: 10.3389/fimmu.2019.02478 (PMC6851059; doi:10.3389/fimmu.2019.02478)
Supplement: Supplementary file 1 [file Data_Sheet_1.zip › Figures/Figure 5.pdf]

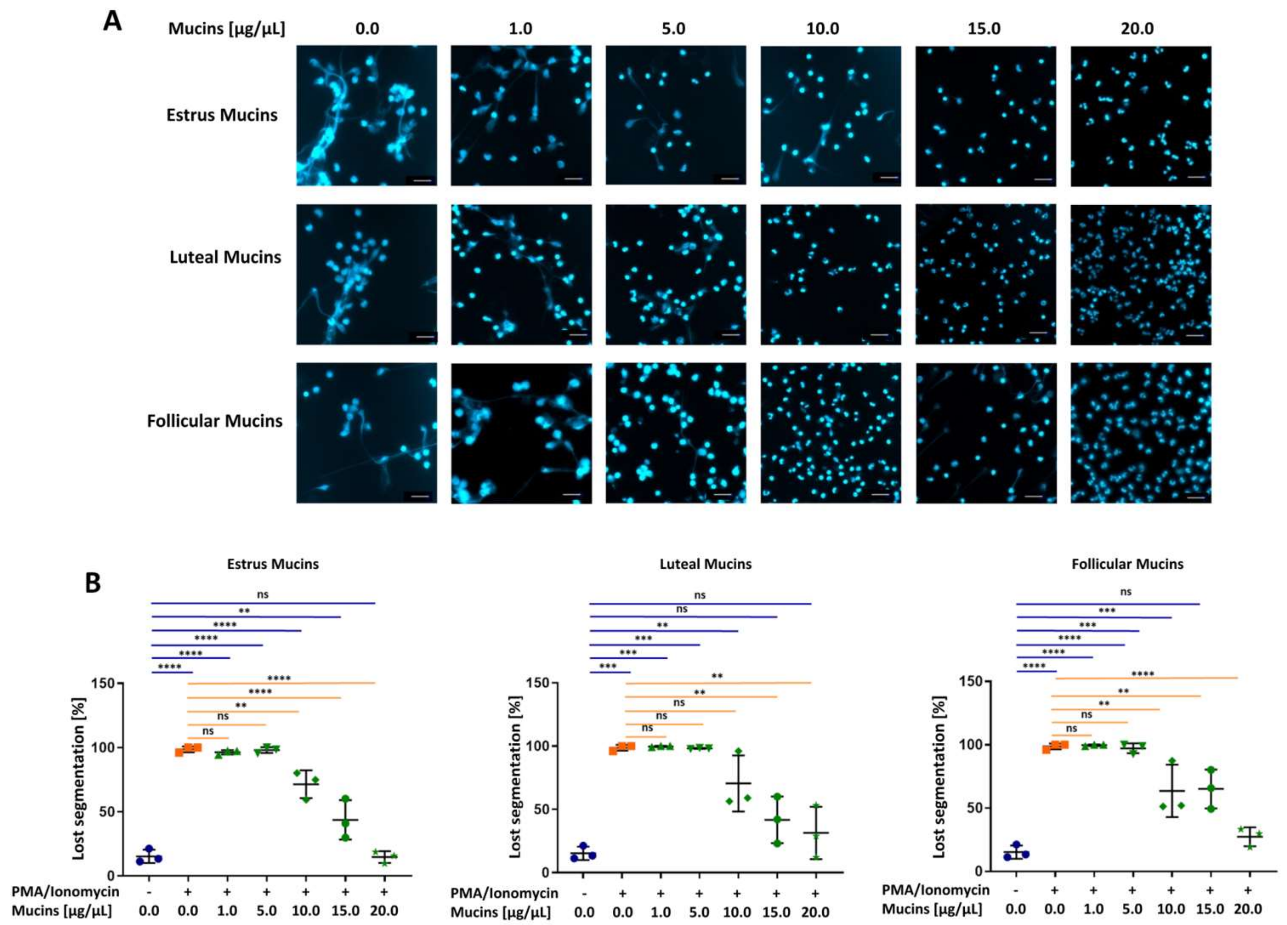

Supplementary Figure 5. Mucins prevent NET release induced by a combination of PMA and ionomycin in a concentration dependent manner. A) Different concentrations of bovine cervical mucins were added to neutrophils stimulated with 1.5 µM PMA in combination with 3 µM ionomycin for 4 h. DNA was stained with DAPI. Pictures of NET inhibition by the addition of 20 µg/µL mucins are also shown in Figure 4. B) Total cell number and cells with remaining segmented nuclei were counted and the percentage of activated neutrophils was calculated. Mean values and standard deviations are displayed in the diagrams (n = 3 different animals). Paired ANOVA and a multiple-comparison Tukey test were applied. Statistically significant differences are given: ns, not significant; $^{**}p \leq 0.01$; $^{***}p \leq 0.001$; $^{****}p \leq 0.0001$.
