# Supplementary material for: Sialylated Cervical Mucins Inhibit the Activation of Neutrophils to Form Neutrophil Extracellular Traps in Bovine in vitro Model
Source: Front Immunol. 2019 Nov 6;10:2478. doi: 10.3389/fimmu.2019.02478 (PMC6851059; doi:10.3389/fimmu.2019.02478)
Supplement: Supplementary file 1 [file Data_Sheet_1.zip › Figures/Figure 7.pdf]

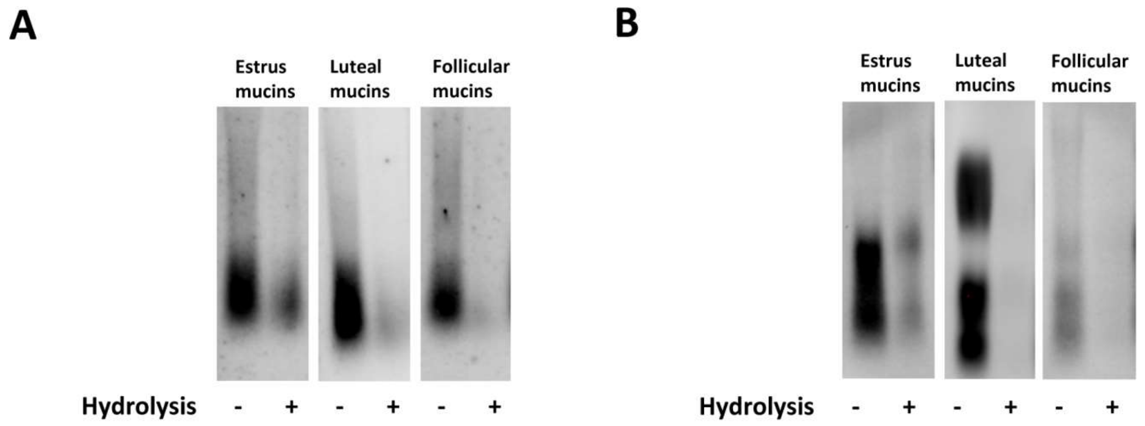

Supplementary Figure 7. Hydrolysis of bovine cervical mucins using acetic acid. A) In order to confirm successful release of sialic acid residues, we performed agarose gels and stained blotted PVDF membranes using the lectin MAL II; staining prevalently α2,3 linked sialic acid and to a weaker extent α2,6 linked sialic acid. B) In order to confirm successful hydrolysis of sialic acid, we performed agarose gels and stained blotted PVDF membranes with the lectin SNA; visualizing mainly α2,6 linked sialic acid. Each three independent experiments were performed.
