# Supplementary material for: Sialylated Cervical Mucins Inhibit the Activation of Neutrophils to Form Neutrophil Extracellular Traps in Bovine in vitro Model
Source: Front Immunol. 2019 Nov 6;10:2478. doi: 10.3389/fimmu.2019.02478 (PMC6851059; doi:10.3389/fimmu.2019.02478)
Supplement: Supplementary file 1 [file Data_Sheet_1.zip › Figures/Figure 9.pdf]

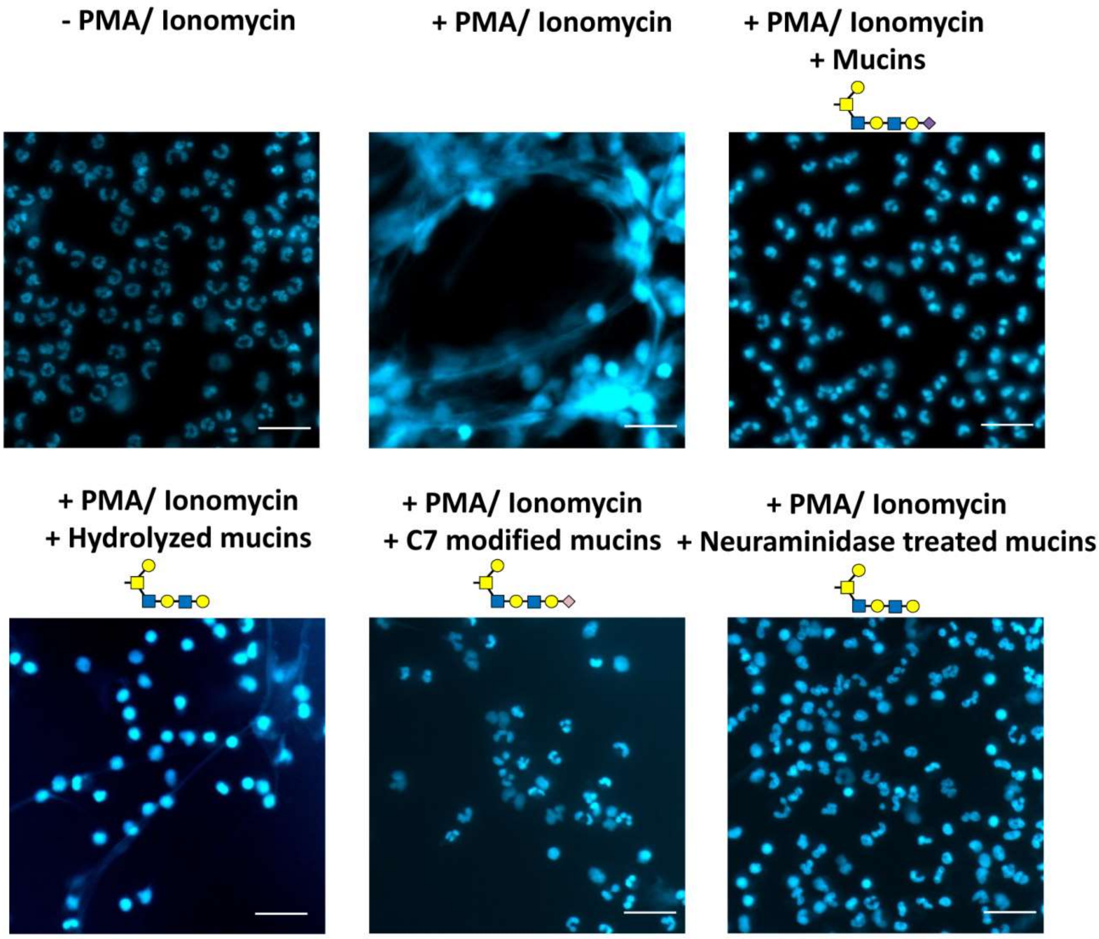

Supplementary Figure 9. Follicular mucins inhibit the release of NETs induced by 1.5 µM PMA in combination with 3 µM ionomycin by sialic acid on its surface. Untreated estrus mucins, as well as hydrolyzed, C7 modified and neuraminidase treated mucins were applied to stimulated neutrophils in a final concentration of 20 µg/µL. DAPI staining was performed. Scale bars: 20 µm. Yellow square: N-acetylgalactosamine, Blue square: N-acetylglucosamine, Yellow circle: Galactose, Purple diamond: Sialic acid, rose diamond: C7 modified sialic acid.
